# Bis[4-chloro-2-(imino­meth­yl)phenolato]copper(II)

**DOI:** 10.1107/S1600536809007624

**Published:** 2009-03-06

**Authors:** Chunbao Tang

**Affiliations:** a Department of Chemistry, Jiaying University, Meizhou 514015, People’s Republic of China

## Abstract

In the title mononuclear copper(II) complex, [Cu(C_7_H_5_ClNO)_2_], the Cu atom, situated on an inversion center, is four-coordinated, in a slightly distorted square-planar geometry, by the N- and O-donor atoms of two symmetry-related 4-chloro-2-(imino­meth­yl)phenolate Schiff base ligands.

## Related literature

For the isotypic Ni(II) complex, see: Hong (2009 ▶). For bio-inorganic chemistry and the coordination chemistry of copper(II) complexes, see: Datta *et al.* (2008 ▶); Diallo *et al.* (2008 ▶); Khalaji *et al.* (2009 ▶).
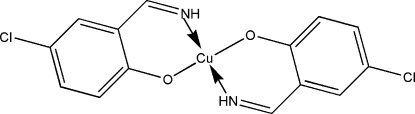


## Experimental

### 

#### Crystal data

[Cu(C_7_H_5_ClNO)_2_]
*M* *_r_* = 372.68
Monoclinic, 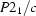

*a* = 15.775 (4) Å
*b* = 5.6949 (14) Å
*c* = 7.886 (2) Å
β = 93.932 (3)°
*V* = 706.8 (3) Å^3^
*Z* = 2
Mo *K*α radiation
μ = 1.93 mm^−1^
*T* = 298 K
0.18 × 0.17 × 0.17 mm

#### Data collection

Bruker SMART CCD area-detector diffractometer
Absorption correction: multi-scan (*SADABS*; Sheldrick, 1996 ▶) *T*_min_ = 0.723, *T*_max_ = 0.735
3835 measured reflections
1488 independent reflections
1025 reflections with *I* > 2σ(*I*)
*R*_int_ = 0.038

#### Refinement

*R*[*F* ^2^ > 2σ(*F* ^2^)] = 0.047
*wR*(*F* ^2^) = 0.139
*S* = 1.01
1488 reflections
97 parameters
H-atom parameters constrained
Δρ_max_ = 0.48 e Å^−3^
Δρ_min_ = −0.57 e Å^−3^

### 

Data collection: *SMART* (Bruker, 2002 ▶); cell refinement: *SAINT* (Bruker, 2002 ▶); data reduction: *SAINT*; program(s) used to solve structure: *SHELXS97* (Sheldrick, 2008 ▶); program(s) used to refine structure: *SHELXL97* (Sheldrick, 2008 ▶); molecular graphics: *SHELXTL* (Sheldrick, 2008 ▶); software used to prepare material for publication: *SHELXTL*.

## Supplementary Material

Crystal structure: contains datablocks global, I. DOI: 10.1107/S1600536809007624/su2100sup1.cif

Structure factors: contains datablocks I. DOI: 10.1107/S1600536809007624/su2100Isup2.hkl

Additional supplementary materials:  crystallographic information; 3D view; checkCIF report

## Figures and Tables

**Fig. 1.**
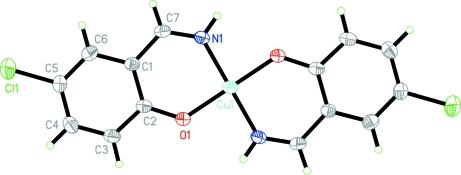
The structure of the title compound, showing the atom-numbering scheme and displacement ellipsoids drawn at the 30% probability level.

## supplementary crystallographic information

### Comment

Copper(II) complexes have been widely investigated in both bioinorganic chemistry and coordination chemistry (Diallo *et al.*, 2008; Datta *et al.*, 2008; Khalaji *et al.*, 2009). As a further study of the structures of such complexes, the crystal structure of the title mononuclear copper(II) complex is reported here. The title complex is isostructural with the nickel(II) complex of the same ligand, 4-Chloro-2-(iminomethyl)phenolate, reported on recently by (Hong, 2009).

The molecular structure of the title complex is illustrated in Fig. 1, and geometrical parameters are given in the archived CIF. The Cu^II^ atom lies on an inversion center and is four-coordinated in a square-planar geometry by the N-and O-donor atoms of two Schiff base ligands. The whole molecule of the complex is approximately coplanar with mean deviation from the least-squares plane of 0.021 (2) Å.

### Experimental

5-Chloro-2-hydroxybenzaldehyde (0.2 mmol, 31.3 mg), copper(II) acetate monohydrate (0.1 mmol, 20.0 mg) and three drops of ammonia (30%) were mixed in 10 ml of methanol. The final solution was stirred for 10 min and allowed to stand in air for two days, yielding blue needle-like crystals of the title compound.

### Refinement

The H-atoms were included in calculated positions and treated as riding: C-H = 0.93 Å, N-H = 0.86 Å, and *U*_iso_(H) = 1.2*U*_eq_(C,*N*).

### Crystal data

**Table:**

[Cu(C_7_H_5_ClNO)_2_]	*F*(000) = 374
*M**_r_* = 372.68	*D*_x_ = 1.751 Mg m^−^^3^
Monoclinic, *P*2_1_/*c*	Mo *K*α radiation, λ = 0.71073 Å
*a* = 15.775 (4) Å	Cell parameters from 811 reflections
*b* = 5.6949 (14) Å	θ = 2.5–24.3°
*c* = 7.886 (2) Å	µ = 1.93 mm^−^^1^
β = 93.932 (3)°	*T* = 298 K
*V* = 706.8 (3) Å^3^	Cut from needle, blue
*Z* = 2	0.18 × 0.17 × 0.17 mm

### Data collection

**Table:**

Bruker SMART CCD area-detector diffractometer	1488 independent reflections
Radiation source: fine-focus sealed tube	1025 reflections with *I* > 2σ(*I*)
graphite	*R*_int_ = 0.038
ω scans	θ_max_ = 26.7°, θ_min_ = 2.6°
Absorption correction: multi-scan (*SADABS*; Sheldrick, 1996)	*h* = −19→19
*T*_min_ = 0.723, *T*_max_ = 0.735	*k* = −7→4
3835 measured reflections	*l* = −9→9

### Refinement

**Table:**

Refinement on *F*^2^	Primary atom site location: structure-invariant direct methods
Least-squares matrix: full	Secondary atom site location: difference Fourier map
*R*[*F*^2^ > 2σ(*F*^2^)] = 0.047	Hydrogen site location: inferred from neighbouring sites
*wR*(*F*^2^) = 0.139	H-atom parameters constrained
*S* = 1.01	*w* = 1/[σ^2^(*F*_o_^2^) + (0.0757*P*)^2^ + 0.0803*P*] where *P* = (*F*_o_^2^ + 2*F*_c_^2^)/3
1488 reflections	(Δ/σ)_max_ < 0.001
97 parameters	Δρ_max_ = 0.48 e Å^−^^3^
0 restraints	Δρ_min_ = −0.57 e Å^−^^3^

### Special details

**Table:**

Geometry. All e.s.d.'s (except the e.s.d. in the dihedral angle between two l.s. planes) are estimated using the full covariance matrix. The cell e.s.d.'s are taken into account individually in the estimation of e.s.d.'s in distances, angles and torsion angles; correlations between e.s.d.'s in cell parameters are only used when they are defined by crystal symmetry. An approximate (isotropic) treatment of cell e.s.d.'s is used for estimating e.s.d.'s involving l.s. planes.
Refinement. Refinement of *F*^2^ against ALL reflections. The weighted *R*-factor *wR* and goodness of fit *S* are based on *F*^2^, conventional *R*-factors *R* are based on *F*, with *F* set to zero for negative *F*^2^. The threshold expression of *F*^2^ > σ(*F*^2^) is used only for calculating *R*-factors(gt) *etc*. and is not relevant to the choice of reflections for refinement. *R*-factors based on *F*^2^ are statistically about twice as large as those based on *F*, and *R*- factors based on ALL data will be even larger.

### Fractional atomic coordinates and isotropic or equivalent isotropic displacement parameters (Å^2^)

**Table:**

	*x*	*y*	*z*	*U*_iso_*/*U*_eq_	
Cu1	0.5000	1.0000	1.0000	0.0394 (3)	
Cl1	0.93518 (8)	0.8488 (3)	0.8060 (2)	0.0767 (5)	
N1	0.5411 (2)	0.7404 (6)	0.8909 (4)	0.0407 (8)	
H1	0.5043	0.6336	0.8631	0.049*	
O1	0.60283 (17)	1.1520 (5)	1.0157 (4)	0.0418 (7)	
C1	0.6875 (3)	0.8577 (7)	0.8857 (5)	0.0361 (9)	
C2	0.6766 (3)	1.0747 (7)	0.9685 (5)	0.0369 (9)	
C3	0.7494 (3)	1.2131 (8)	1.0047 (5)	0.0446 (11)	
H3	0.7444	1.3543	1.0624	0.053*	
C4	0.8280 (3)	1.1444 (8)	0.9568 (6)	0.0506 (12)	
H4	0.8751	1.2397	0.9817	0.061*	
C5	0.8372 (3)	0.9355 (8)	0.8721 (6)	0.0468 (11)	
C6	0.7682 (3)	0.7904 (8)	0.8369 (6)	0.0460 (11)	
H6	0.7749	0.6485	0.7811	0.055*	
C7	0.6171 (3)	0.6979 (7)	0.8509 (5)	0.0411 (10)	
H7	0.6274	0.5568	0.7967	0.049*	

### Atomic displacement parameters (Å^2^)

**Table:**

	*U*^11^	*U*^22^	*U*^33^	*U*^12^	*U*^13^	*U*^23^
Cu1	0.0443 (5)	0.0320 (4)	0.0413 (5)	−0.0014 (3)	−0.0013 (3)	−0.0036 (3)
Cl1	0.0436 (7)	0.0892 (11)	0.0988 (12)	0.0013 (7)	0.0152 (7)	−0.0162 (9)
N1	0.041 (2)	0.0338 (19)	0.047 (2)	−0.0048 (15)	−0.0007 (16)	−0.0067 (15)
O1	0.0398 (17)	0.0346 (17)	0.0509 (18)	−0.0019 (12)	0.0033 (13)	−0.0081 (13)
C1	0.041 (2)	0.031 (2)	0.036 (2)	−0.0007 (17)	−0.0005 (17)	0.0020 (17)
C2	0.046 (3)	0.031 (2)	0.033 (2)	−0.0010 (18)	−0.0024 (18)	−0.0008 (16)
C3	0.051 (3)	0.034 (2)	0.048 (3)	−0.0038 (19)	−0.003 (2)	−0.0040 (18)
C4	0.042 (3)	0.052 (3)	0.057 (3)	−0.008 (2)	−0.001 (2)	0.002 (2)
C5	0.037 (2)	0.052 (3)	0.051 (3)	0.003 (2)	0.004 (2)	0.001 (2)
C6	0.051 (3)	0.040 (3)	0.048 (3)	0.006 (2)	0.004 (2)	0.0001 (19)
C7	0.050 (3)	0.030 (2)	0.042 (2)	0.0008 (18)	−0.0004 (19)	−0.0048 (18)

### Geometric parameters (Å, °)

**Table:**

Cu1—O1^i^	1.835 (3)	C1—C7	1.447 (6)
Cu1—O1	1.835 (3)	C2—C3	1.406 (6)
Cu1—N1^i^	1.850 (3)	C3—C4	1.378 (6)
Cu1—N1	1.850 (3)	C3—H3	0.9300
Cl1—C5	1.736 (5)	C4—C5	1.377 (7)
N1—C7	1.282 (5)	C4—H4	0.9300
N1—H1	0.8600	C5—C6	1.380 (6)
O1—C2	1.321 (5)	C6—H6	0.9300
C1—C6	1.408 (6)	C7—H7	0.9300
C1—C2	1.413 (6)		
			
O1^i^—Cu1—O1	180.00 (8)	C4—C3—C2	121.6 (4)
O1^i^—Cu1—N1^i^	94.10 (14)	C4—C3—H3	119.2
O1—Cu1—N1^i^	85.90 (14)	C2—C3—H3	119.2
O1^i^—Cu1—N1	85.90 (14)	C5—C4—C3	120.3 (4)
O1—Cu1—N1	94.10 (14)	C5—C4—H4	119.8
N1^i^—Cu1—N1	180.000 (1)	C3—C4—H4	119.8
C7—N1—Cu1	128.9 (3)	C4—C5—C6	120.5 (4)
C7—N1—H1	115.5	C4—C5—Cl1	121.1 (4)
Cu1—N1—H1	115.5	C6—C5—Cl1	118.4 (4)
C2—O1—Cu1	128.0 (3)	C5—C6—C1	119.9 (4)
C6—C1—C2	120.3 (4)	C5—C6—H6	120.1
C6—C1—C7	118.3 (4)	C1—C6—H6	120.1
C2—C1—C7	121.4 (4)	N1—C7—C1	123.5 (4)
O1—C2—C3	118.6 (4)	N1—C7—H7	118.2
O1—C2—C1	124.0 (4)	C1—C7—H7	118.2
C3—C2—C1	117.4 (4)		

Symmetry codes: (i) −*x*+1, −*y*+2, −*z*+2.
